# Role of Surface-Active Element Sulfur on Thermal Behavior, Driving Forces, Fluid Flow and Solute Dilution in Laser Linear Welding of Dissimilar Metals

**DOI:** 10.3390/ma16072609

**Published:** 2023-03-25

**Authors:** Zhuang Shu, Gang Yu, Binxin Dong, Xiuli He, Zhiyong Li, Shaoxia Li

**Affiliations:** 1 Institute of Mechanics, Chinese Academy of Sciences, Beijing 100190, China; shuzhuang@imech.ac.cn (Z.S.); gyu@imech.ac.cn (G.Y.); dongbinxin@imech.ac.cn (B.D.); lisx@imech.ac.cn (S.L.); 2 School of Engineering Science, University of Chinese Academy of Sciences, Beijing 100190, China; 3 Center of Materials Science and Optoelectronics Engineering, University of Chinese Academy of Sciences, Beijing 100190, China; 4 Guangdong Aerospace Research Academy, Guangzhou 511458, China

**Keywords:** laser dissimilar welding, surface-active element, driving forces, heat transfer, mass transfer

## Abstract

Understanding heat and mass transfer and fluid flow in the molten pool is very helpful in the selection and optimization of processing parameters, and the surface-active element has an important effect on the heat and mass transfer in laser welding of dissimilar metals. A three-dimensional (3D) numerical model coupled with a sub-model of surface tension, which considers the influence of local temperature and the concentration of surface-active element sulfur at the gas/liquid surface, is used to analyze the thermal behavior, driving forces, fluid flow, and solute dilution during laser linear welding of 304SS and Ni. The relationship between surface tension, driving forces, and the temperature coefficient of surface tension with the spatial distribution of temperature and the surface-active element sulfur is quantitatively analyzed. The simulation results show that the molten pool is fully developed at 45 ms, and the collision of inward and outward convection, with the maximum velocity reaching 1.7 m/s, occurs at the isotherm with a temperature between 2200 K and 2500 K. The temperature-gradient term and concentration-gradient term of surface shear stress play different roles in different positions of the free surface. The local sulfur concentration changes the temperature sensitivity of the surface tension at different sides of the free surface and further determines the transition of convection. Complex fluid flow promotes solute dilution, and the distribution of solute becomes uniform from the front to the rear of the molten pool. The Ni element is transferred to 304SS mainly at the rear side. The work provides theoretical support for the control of joint quality by changing the content of surface-active elements in dissimilar welding.

## 1. Introduction

Welded connections between dissimilar metals have gained widespread attention because of their flexibility, low cost, and excellent performance, which are of significant importance for both scientific research and engineering applications [1,2,3,4,5,6]. For instance, the combination of expensive, corrosion-resistant pure nickel and less expensive, heat-resistant 304 stainless steel is commonly used in petrochemical, steel metallurgy, and aerospace applications [7,8]. Due to its high power density, high precision, high efficiency, and low distortion, laser welding is widely used in the jointing of dissimilar metals [1,9,10,11,12,13]. However, owing to the differences in composition and thermophysical properties of the base metals, defects, e.g., improper mixing of alloying elements and the formation of brittle intermetallic phases, still occur. Experimental observation of high-temperature fluid flow in the molten pool is difficult. Numerical simulation is an effective tool to elucidate the mechanism of heat and mass transfer, which facilitates the optimization and improvement of weld quality.

Numerical models for laser welding have been fully developed over the past few decades, leading to substantial progress in understanding the driving mechanisms of heat transfer and fluid flow [14,15,16,17,18,19]. He et al. [14] studied the heat transfer mechanism by the Peclet number in the laser spot welding and concluded, by the comparison of the dimensionless numbers Ma to Gr, that surface tension played a dominant role in driving the liquid flow. Wang et al. [15] proposed that the recoil pressure was the primary driving force that pushed down the liquid in the molten pool during the laser keyhole welding. Through a laser spot welding model, Saldi et al. [16] explored the effect of the cooling stage on the profile of the weld and concluded that the fluid flow could still alter the weld morphology after the pulsed laser was turned off, owing to the temperature-dependent surface tension. To investigate the heat and mass transfer during the laser linear welding, an improved 3D model was developed by Li et al. [17], and it was found that the main pattern of mass transportation was convection, with a Peclet number for mass transfer that reached the order of 1000. Duggirala et al. [19] studied the deep penetration laser welding process and concluded that the flow in the molten pool was laminar, with Marangoni convection being dominant.

In laser welding, fluid flow, which plays a critical role in heat and mass transfer, depends on the surface tension. The relationship between the distribution of temperature and surface tension at the free surface, expressed as the temperature coefficient of surface tension (TCST), will determine the convection pattern. Hu et al. [20] analyzed the relationship between the sign of the TCST, the morphology, and the flow pattern in laser spot welding by using a self-developed model, and they concluded that the positive or negative TCST affected the depth and aspect ratio of the weld. The distribution of concentration of surface-active elements (e.g., sulfur and oxygen) and temperature on the free surface will influence the TCST, and under certain conditions, its sign may change, which will affect the distribution of surface tension, flow pattern, and heat and mass transfer [21,22,23,24]. Ribic et al. [25] studied the influence of surface-active elements during keyhole-mode laser welding and found that the change of the sulfur content in the base metal and the O_2_ concentration in the shielding gas results in the change of the width and depth of the weld. Lienert et al. [26] observed a significant centerline shift in the molten pool during Gas Tungsten Arc (GTA) welding when two stainless steels with greatly different sulfur contents were welded, and they suggested that sulfur-influenced Marangoni convection was one of the reasons. Based on this, Wei et al. [27] analyzed the offset of maximum molten depth and rotation of the molten pool shape, and they concluded that the spatial gradient of temperature and sulfur caused the centerline shift and the rotation of the molten pool. Bahrami et al. [28] studied sulfur-induced driving forces at the free surface in GTA spot welding of 1018 steel and nickel 200, and they indicated that the driving forces induced by the concentration gradient of sulfur mainly affected Marangoni convection when the molten pool was just formed.

From above, most studies were focused on the characteristic of weld pool geometry in the presence of the surface-active sulfur element, which caused the change in the sign of the TCST and thus affected the Marangoni flow in the molten pool. However, the thermal behavior, fluid flow, and resultant mass transfer under the action of sulfur dilution in laser welding still need to be investigated systematically. Furthermore, the content of sulfur was changing continuously, driven by the fluid flow in the molten pool. As a result, the relationship between the surface-active element, TCST, surface tension, and fluid flow becomes more complex. Therefore, the quantitative analysis of sulfur-induced heat and mass transfer in laser welding is necessary for understanding the solidification mechanism and optimizing the process for desired component, microstructure, and mechanical properties.

In this study, the relationship between the heat and mass transportation and driving forces in laser linear welding of 304SS and Ni is systematically investigated. A 3D numerical model coupled with a sub-model of surface tension under the influence of local temperature and sulfur content is used. First, the thermal behavior and the evolution of the molten pool are analyzed. Next, the relationship between the dilution of sulfur on the free surface and driving forces is quantitatively investigated. Finally, the fluid flow and mass transfer considering the sulfur-induced flow transition are studied and verified with the results of EDS.

## 2. Numerical Model

**Figure 1:**
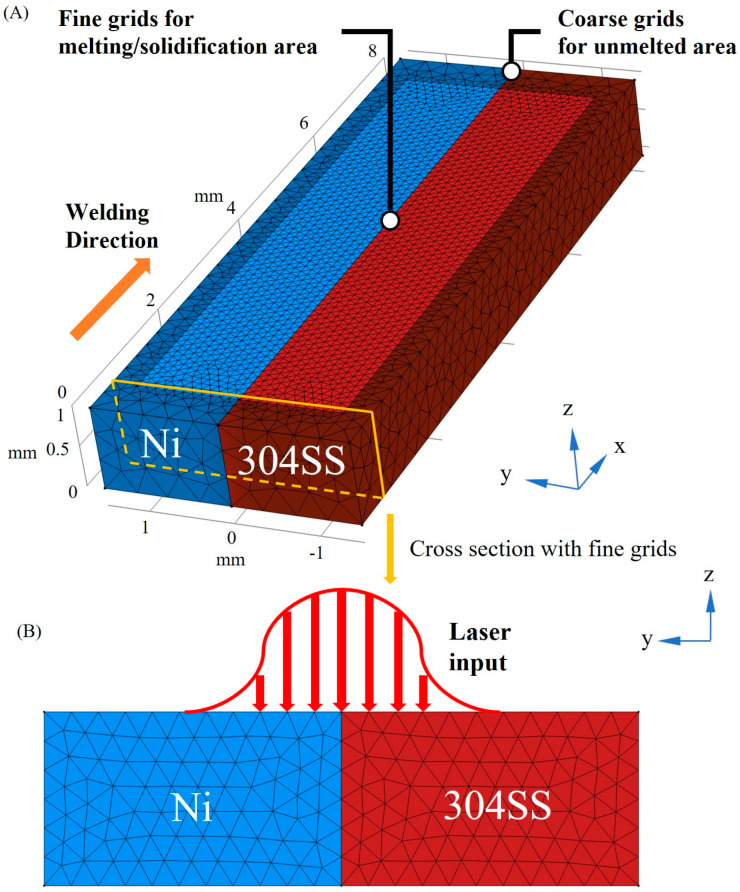
Grid distribution in the numerical model. (**A**) 3D view; (**B**) Cross-section with fine grids.

The geometry and discretization in the numerical model for laser welding of 304SS and Ni are plotted in Figure 1, with fine grids in the region where the melting/solidification process occurs and coarse grids in the unmelted region. The chemical composition of 304SS is presented in Table 1, and the Ni substrate is assumed to be pure nickel. The thermophysical properties of 304SS and Ni are presented in Table 2. In this study, the following assumptions are used to simplify this model:

1. The liquid metal in the molten pool is an incompressible, Newtonian, and laminar flow.
2. The laser power distribution is Gaussian.
3. The free surface is set as a flat plane. The role of buoyancy is described by the Boussinesq approximation.
4. Material properties are temperature-independent.

### 2.1. Governing Equations

Based on the conservation equations for mass, momentum, energy, and solute transfer and the above assumptions, the governing equations are expressed in Equations (1)–(4):

(1) $$\frac{\partial \rho }{\partial t}+\nabla \cdot \left(\rho u\right)=0$$

(2) $$\rho \frac{\partial u}{\partial t}+\rho (u\cdot \nabla )u=\nabla \cdot [-pI+\mu (\nabla u+{\left(\nabla u\right)}^{T})-\frac{2}{3}u(\nabla u)I]+F_{d}+F_{b}$$

(3) $$\rho C_{P}\frac{\partial T}{\partial t}+\rho C_{P}u\cdot \nabla T=\nabla \cdot (k\nabla T)-\rho \frac{\partial (\Delta H)}{\partial t}-\rho u\cdot \nabla (\Delta H)$$

(4) $$\frac{\partial C_{i}}{\partial t}+u\cdot \nabla C_{i}=\nabla \cdot (D_{i}\nabla C_{i})$$

where *ρ* is the density, *t* is the time variable, ***u*** is the velocity, *μ* is the viscosity, *p* is the pressure, *T* is the temperature, *k* is the thermal conductivity, *C_P_* is the specific heat, and *D_i_* and *C_i_* are the *i*th component of the diffusion factor and the concentration, respectively.

The fourth term on the right-hand side of Equation (2) is used to represent the resistance of porous media in the mushy zone based on the Carmen-Kozeny hypothesis, which is described in Equation (5).

(5) $$F_{d}=-A_{\mathrm{mush}}u{\left(1-f_{l}\right)}^{2}/(f_{l}^{3}+M)$$

In Equation (5), *A_mush_* represents the resistance of the mushy zone, which is set to 10^7^ kg/m^3^·s in this study, *M* is a small number to avoid the division by zero, and *f_l_* is the liquid fraction expressed as:

(6) $$f_{l}=\left\{ \begin{array}{cc} 1, & \;T>T_{l}\\ \frac{T-T_{s}}{T_{l}-T_{s}}, & \;T_{s}<T<T_{l}\\ 0, & \;0<T<T_{s} \end{array}\right.$$

where *T_l_* and *T_s_* are the liquidus and solidus temperatures, respectively.

The fifth term on the right-hand side of Equation (2) is used to represent the volume forces due to changes in temperature and concentration, which are described in Equation (7):

(7) $$F_{b}=-\rho \left[\beta_{T}\left(T-T_{\mathrm{ref}}\right)+\beta_{C}\left(C-C_{\mathrm{ref}}\right)\right]g$$

where *β_T_* and *β_C_* represent the volumetric expansion coefficients caused by temperature and composition, respectively. *T_ref_* is the ambient temperature, *C* is the local concentration, and *C_ref_* is the reference concentration. Δ*H* in Equation (3) represents the latent heat of phase change, which is described in Equation (8):

(8) $$\Delta H=Lf_{l}$$

where *L* is the latent heat of fusion.

### 2.2. Sub-Model of Surface Tension

Convection is mainly driven by surface tension in laser welding [14], and the TCST is affected by both the local temperature and content of the surface-active element (sulfur in this study). The initial sulfur concentration is set at 0.0072% in 304SS and 0.0003% in pure nickel. The functions describing the variations of surface tension and TCST versus local temperature and sulfur content are proposed by Sahoo et al. [23] and can be given as follows:

(9) $$\gamma \left(T\right)=\gamma_{p}-A\left(T-T_{l}\right)-\mathrm{RT}\Gamma_{S}\ln\left(1+K\alpha_{S}\right)$$

(10) $$\frac{\partial \gamma }{\partial T}=-A-R\Gamma_{S}\ln(1+K\alpha_{S})-\frac{K\alpha_{S}}{1+K\alpha_{S}}\cdot \frac{\Gamma_{S}\Delta H^{0}}{T}$$

(11) $$K=k_{l}\exp(-\frac{\Delta H^{0}}{RT})$$

where *γ_p_* is the surface tension of the pure melting metal, *A* is the negative of TCST, *Γ_S_* is the surface excess at saturation, *R* is the universal gas constant, *K* is the adsorption coefficient, *k_l_* is the constant corresponding to the segregation entropy, Δ*H*^0^ is the standard heat of adsorption, and *α_s_* is the activity of the surface-active element, which could be approximated by the weight % of sulfur in the molten pool. When the surface-active element is negligible, the value of *α_s_* is 0, and the TCST is a negative constant. The parameters used in the sub-model of surface tension are given in Table 3. In this study, the value of the TCST varies with the spatial distribution of sulfur concentration and the local temperature.

### 2.3. Boundary Conditions

The laser flux absorbed and the energy loss of the specimen are described as follows:

(12) $$q_{ener}=\frac{2Q_{laser}\eta }{\pi r_{e}^{2}}\exp(-\frac{2r^{2}}{r_{e}^{2}})-h_{c}(T-T_{0})-\sigma_{b}\varepsilon (T^{4}-T_{0}^{4})$$

The first term on the right side represents the energy absorbed from the laser beam with a Gaussian distribution: *Q_laser_* is the laser power, *η* is the absorptivity of laser energy, *r_e_* is the effective radius of the laser spot, and *r* is the distance from the laser center. The second and third terms on the right side represent the heat loss by convection and radiation, respectively. *T_0_* is the ambient temperature, *h_c_* and *σ_b_* are the convection heat transfer coefficient and Stefan-Boltzmann constant, respectively, and *ε* is the emissivity. The welding parameters used in the simulation are listed in Table 4, and the center of the laser beam is located at the contact position of 304SS and Ni.

Since the free surface is set as a plane, the thermocapillary force mainly drives the convection in the molten pool [14], and momentum boundary conditions are described as follows:

(13) $$\mu \frac{\partial u}{\partial z}=f_{l}\frac{d\gamma }{dT}\frac{\partial T}{\partial x}+f_{l}\frac{d\gamma }{dC_{s}}\frac{\partial C_{s}}{\partial x}$$

(14) $$\mu \frac{\partial v}{\partial z}=f_{l}\frac{d\gamma }{dT}\frac{\partial T}{\partial y}+f_{l}\frac{d\gamma }{dC_{s}}\frac{\partial C_{s}}{\partial y}$$

where *u* and *v* are the velocities, and *C_s_* is the concentration of sulfur.

Since there is no mass addition, the boundary condition for the solute transport equation can be expressed as follows:

(15) $$(-D_{Ci}\nabla C_{i}+C_{i}u_{Ci})\cdot n=0$$

where *D_Ci_* is the coefficient of diffusivity and ***u_Ci_*** is the velocity of the mass flux for one certain element.

### 2.4. Model Verification

The model verification was performed by comparing the simulated and experimental results. Laser joining experiments were performed using a Nd:YAG laser beam on a program-controlled five-axis laser manufacturing system. Before the experiment, the impurities on the sample surface were removed by using acetone. The prepared samples were then subjected to a series of treatments, including mounting, polishing, and etching in an aqua regia solution that prepares metallurgical samples. The treated specimens were eventually observed and measured using an optical microscope (OM), a ZEISS EV18 scanning electron microscope (SEM), and an Oxford INCA energy dispersive spectrometer (EDS).

## 3. Results and Discussion

### 3.1. Thermal Behavior

**Figure 2:**
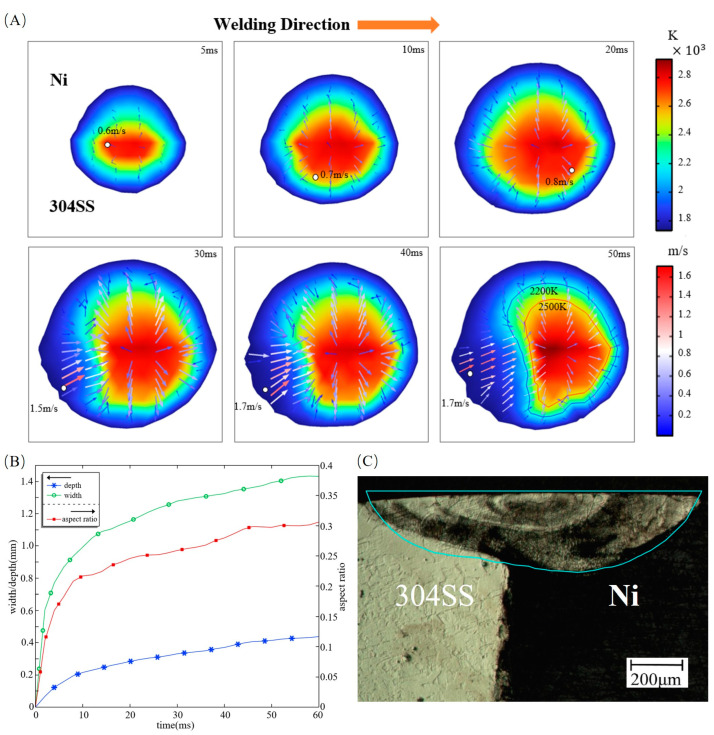
Evolution of the temperature field, the velocity field, and the dimensions and morphology of the molten pool. (**A**) Evolution of the temperature field and velocity field; (**B**) Dimensions of the molten pool; (**C**) Comparison between the simulated and experimental [17] cross-sectional profiles. Parameters: laser radius of 0.57 mm, welding speed of 20 mm/s, and laser power of 800 W.

In laser welding, the morphological characteristics of the joint are significantly influenced by the thermal behavior. Figure 2A,B demonstrate the evolution of the temperature and velocity fields, as well as the dimensions of the molten pool, respectively. The molten pool is formed due to the absorption of laser energy by base metals. In the early stage of molten pool formation, outward convection is dominant for the fluid flow at the free surface, with a maximum velocity of 0.8 m/s at the periphery of the free surface. After 20 ms, there is a strong inward convection at the rear of the free surface on the 304SS side, which is at an angle to the welding direction. Its maximum velocity of 1.7 m/s is approximately twice the maximum velocity of the outward convection, which is located in the region of flow from the center towards the tail and has a magnitude of 0.9 m/s. The inward convection collides with the outward convection at positions where the temperature is between 2200 K and 2500 K.

To illustrate the heat transfer mechanism with the evolution of the molten pool, the Peclet number, which represents the relative importance of heat conduction to heat convection, is introduced in Equation (16):

(16) $$Pe_{T}=\frac{U_{\max}L}{\alpha }$$

where *U_max_* is the maximum fluid flow velocity, *L* is the characteristic length, which is equal to the molten pool depth in this study, and *α* is the thermal diffusivity, which is defined as *k/*(*C_P_·ρ*). At 5 ms, *Pe_T_* on the 304SS side is 20 and 4 on the Ni side. This indicates that the dominant mechanism of heat transfer on the 304SS side is already thermal convection during the initial stage of molten pool formation, while the contribution of heat conduction on the Ni side cannot be neglected. As plotted in Figure 2B, the molten pool dimensions increase rapidly before 20 ms, and the aspect ratio, which is defined by the ratio of the maximum width to the maximum depth, amounts to 0.24. Subsequently, the growth rates of pool depth and pool width slow down, and the aspect ratio is stabilized around 0.3 after 45 ms. At this time, the molten pool has been fully developed, and the *Pe_T_* number is 400 for the 304SS side and 60 for the Ni side, which indicates that thermal convection determines heat transfer in the molten pool. The simulated and experimental cross-sectional profiles [17] are compared in Figure 2C, which shows good agreement in terms of size and morphology.

### 3.2. Dilution of Sulfur and Driving Forces

Fluid flow in the molten pool is mainly driven by the surface tension and, to a lesser degree, by the buoyancy force [14]. In this study, the temperature gradient together with the concentration gradient of the sulfur determines the surface shear stress, as illustrated in Equations (13) and (14). To understand the driving mechanism of the liquid metal flow in laser linear welding of 304SS with relatively higher sulfur and nickel with extremely low sulfur, the mass transportation of sulfur is first investigated.

**Figure 3:**
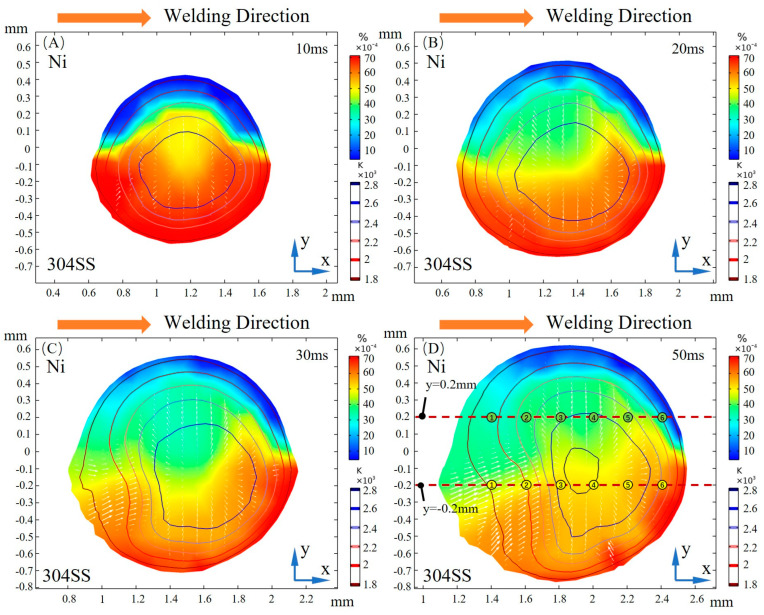
Dilution of sulfur on the free surface at different times. (**A**) 10 ms; (**B**) 20 ms; (**C**) 30 ms; (**D**) 50 ms.

Figure 3 illustrates the dilution of sulfur at the free surface at different times. During the early stage of molten pool formation, the sulfur concentration resembles the solute distribution at the beginning of spot welding [8,28]. Before 30 ms, the transport of elemental sulfur to the Ni side occurs mainly at the front of the molten pool. This is due to the front just melting while the rear tends to solidify before sufficient dilution, which is induced by the movement of the laser. When the molten pool is fully developed, a region with a uniform distribution of sulfur is formed where the temperature is beyond 2200 K. At the rear of the molten pool, the mixing is almost complete, accompanied by a significant gradient distribution in the y direction and a slight variation of 0.001% weight fraction along the x direction.

**Figure 4:**
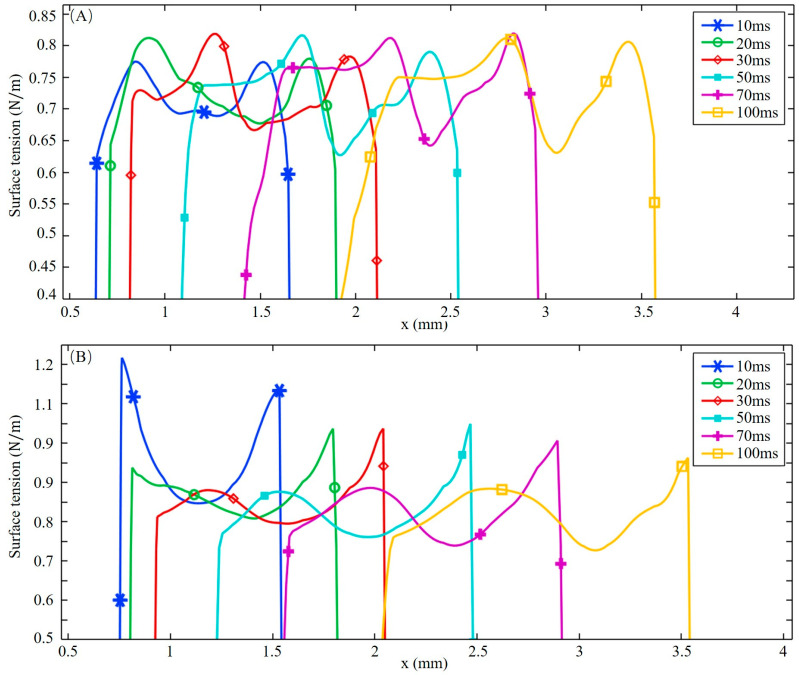
Surface tension distribution at different times. (**A**) y = −0.2 mm; (**B**) y = 0.2 mm.

Figure 4 shows the surface tension at different times. Two lines plotted in Figure 3D, y = −0.2 mm and y = 0.2 mm, are chosen to quantitatively study the characteristics of surface tension when considering sulfur. At the early stage of molten pool development, the sulfur is not sufficiently mixed, and the distribution of surface tension is significantly different from that at a quasi-steady state. On the 304SS side, the surface tension rises rapidly at the edge of the molten pool. After reaching about 0.8 N/m, it gradually decreases from the outer to the inner side before 20 ms have passed. This is due to the high sulfur content in 304SS, which leads to a positive TCST in the low temperature region and a negative value with increasing temperature. After 30 ms, the region with a positive TCST becomes larger due to heat accumulation at the rear of the molten pool. Before 20 ms, the surface tension rises from the center to the outside on the Ni-side free surface, which is caused by a negative TCST due to the low sulfur concentration in the nickel. After 30 ms, from the rear to the front of the free surface, the surface tension increases, then decreases, and then increases again as a result of the solute mixing and dilution process at the rear of the free surface, as plotted in Figure 3C. The coupling coefficient between surface tension and temperature becomes positive here, considering the effect of sulfur. After 50 ms, the distribution of surface tension has stabilized.

**Figure 5:**
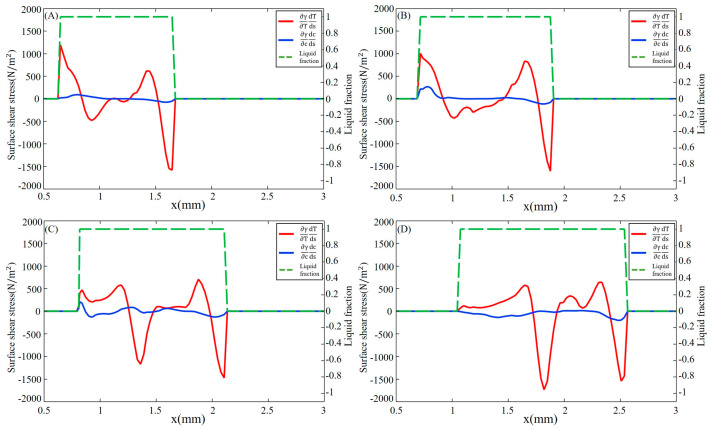
Surface shear stress in the longitudinal section with y = −0.2 mm on the 304SS side at different times. (**A**) 10 ms; (**B**) 20 ms; (**C**) 30 ms; (**D**) 50 ms.

**Figure 6:**
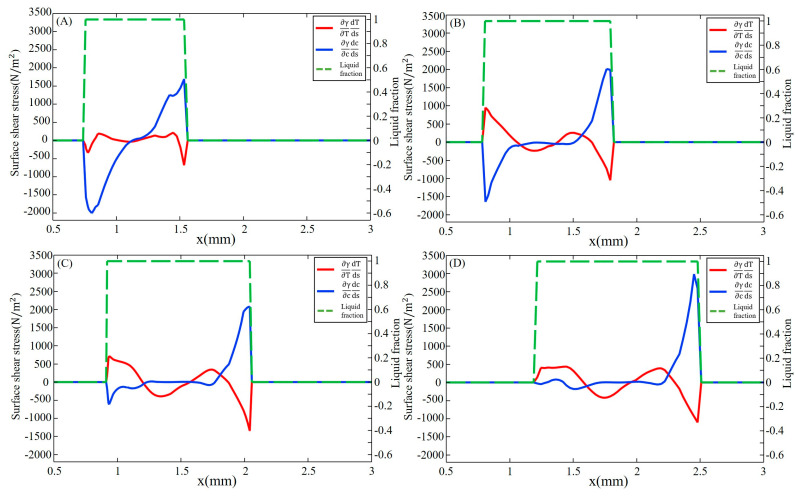
Surface shear stress in the longitudinal section with y = 0.2 mm on the Ni side at different times. (**A**) 10 ms; (**B**) 20 ms; (**C**) 30 ms; (**D**) 50 ms.

The surface shear stress induced by sulfur-influenced surface tension can be decomposed into a temperature-gradient term $\frac{\partial \gamma }{\partial T}\frac{\partial T}{\partial s}$ and a concentration-gradient term $\frac{\partial \gamma }{\partial C}\frac{\partial C}{\partial s}$ as described in Equations (13) and (14). The distribution of surface shear stress at y = −0.2 mm and y = 0.2 mm, marked in Figure 3D, is shown in Figure 5 and Figure 6, respectively. The positive sign for surface shear stress means that the force direction is the same as the welding direction, and the negative sign is the opposite. Due to the temperature and concentration gradient between the substrate and molten pool, a sharp change in surface shear stress is observed near the molten pool boundary. For the fully developed molten pool, the sharp variation only appears at the front of the mushy zone, while the solidification region shows a flatter distribution due to the mixed dilution of solute and the lower temperature distribution. After 30 ms, the high-temperature gradient caused by the collision of outward high-temperature convection and inward low-temperature convection leads to a peak in surface shear stress at the middle of the molten pool. At 50 ms, the surface shear stress on the 304SS side is mainly determined by the temperature gradient term. As shown in Figure 5D, the magnitude of the concentration-gradient term is from 0 to 200 N/m^2^, and the magnitude of the temperature-gradient term is up to 1800 N/m^2^ after the molten pool reaches a quasi-steady stage. On the Ni side, the surface shear stress is mainly influenced by the concentration gradient in the early stage due to the difference in concentration between the middle of the molten pool where sulfur is diluted and the edge where the Ni is just melted, with a temperature-gradient term of about 500 N/m^2^ and a concentration-gradient term of about 2000 N/m^2^, as illustrated in Figure 6A. After 50 ms, due to a mixing dilution of the solute, the surface shear stress is determined by the temperature gradient, except for the front, where a large concentration difference between the base metal and the molten pool exists. The isotherm shown in Figure 3D on the Ni side is sparser than that of the 304SS side in the quasi-steady state. This means that the temperature gradient is smaller, so the temperature-gradient term and, thus, the surface shear stress inside the molten pool are smaller.

**Figure 7:**
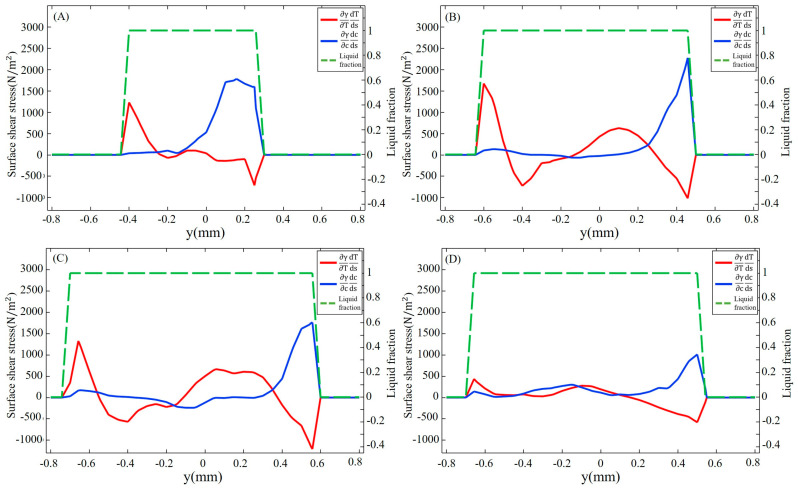
Surface shear stress with x = 1.5 mm at different times. (**A**) 10 ms; (**B**) 20 ms; (**C**) 30 ms; (**D**) 50 ms.

Figure 7 shows the surface shear stress on the yz cross-section at x = 1.5 mm at different times. The symbols indicate the direction of the stress, with positive values indicating the direction from 304SS to Ni and negative values indicating the opposite direction. At 10 ms, the concentration-gradient term of the surface shear stress plays a major role on the Ni side, reaching 1800 N/m^2^, which is three times the peak of the temperature-gradient term, due to the significant difference in sulfur concentration between Ni and 304SS. The surface shear stress on the 304SS side, where the sulfur content in the molten pool is close to that of the base metal, is dominated by the temperature-gradient term. With the continuous dilution of sulfur, the main driving force inside the molten pool becomes the temperature gradient. When the molten pool is solidified, the concentration-gradient term and the temperature-gradient term inside the molten pool are in the same order of magnitude. Under their joint action, the inward convection is deflected toward the centerline of the molten pool, as plotted in Figure 3D.

**Figure 8:**
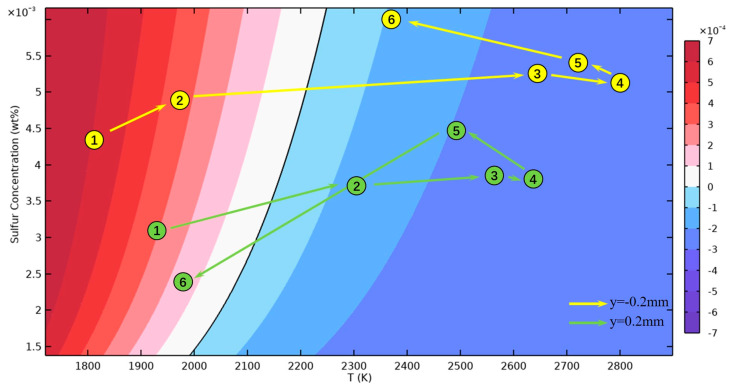
TCST at different temperatures and sulfur concentrations.

To further understand the influence of sulfur on convection, the variation in the TCST is analyzed. The values of the TCST used in this study are plotted in Figure 8. Two lines, y = 0.2 mm and y = −0.2 mm, are chosen for quantitative analysis at 50 ms. The temperature and sulfur content of the uniformly distributed points marked in Figure 3D are plotted in Figure 8 to show the distribution characteristics of the TCST clearly. On the Ni side, the weight fraction of sulfur is between 0.0025% and 0.0045%, and the temperature at which the TCST transitions from a positive signal to a negative signal is around 2100 K. On the 304SS side, the high concentration of sulfur at the molten pool with a weight fraction of 0.0045% to 0.006% makes the TCST sign shift in the higher temperature region of nearly 2200 K, which is closer to the temperature of the molten pool center plotted by the isotherm distribution in Figure 3D. This is the pattern in which the spatial distribution of sulfur and temperature affects the flow.

### 3.3. Fluid Flow

**Figure 9:**
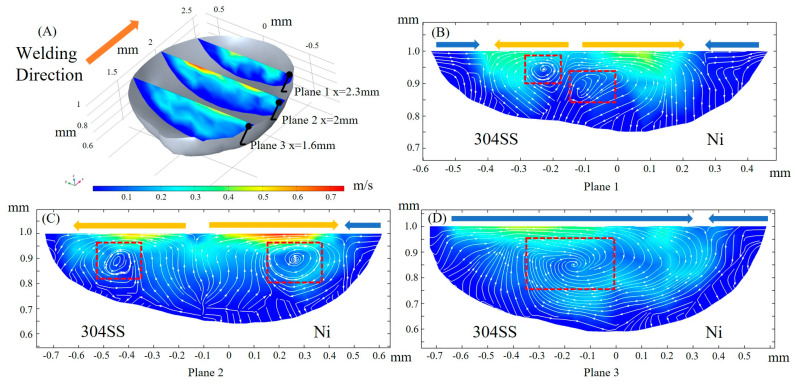
Fluid flow at the yz cross section. (**A**) 3D view; (**B**) x = 2.3 mm; (**C**) x = 2 mm; (**D**) x = 1.6 mm.

The mass transfer is dominated by convection flow in the molten pool [8,17], thus, the fluid flow is analyzed at different planes along the x, y, and z axes. The flow pattern is analyzed when the welding time is 50 ms and the laser is located at x = 2 mm. Figure 9A shows the flow states at the three yz cross sections. Isodensity streamlines are used to represent flow states, and the magnitude of the velocity is denoted by the color map. The yellow arrow above the cross section represents outward convection, and the blue arrow represents inward convection. For plane 1, there are two types of convection, inward and outward, colliding at the molten pool edge. The convection center is located on the 304SS side, and two adjacent vortexes, marked by the red rectangles, exist below the convection center, both located on the 304SS side. This indicates that only a small proportion of the solute in 304SS, such as melted Fe, Cr, etc., is transported to the Ni side. Plane 2 coincides with the center of the laser, and the convection is predominantly outward. With two vortexes moving to either side of the cross section, the distribution of solute in the vicinity of the vortex becomes more homogeneous due to its extended range and stronger fluid flow caused by the large temperature gradient under laser irradiation. However, the dilution of the solutes between the different base metals is still inadequate. For plane 3, there is a large vortex on the rear side of the molten pool, which fills almost the entire cross section and promotes solute mixing between 304SS and Ni.

**Figure 10:**
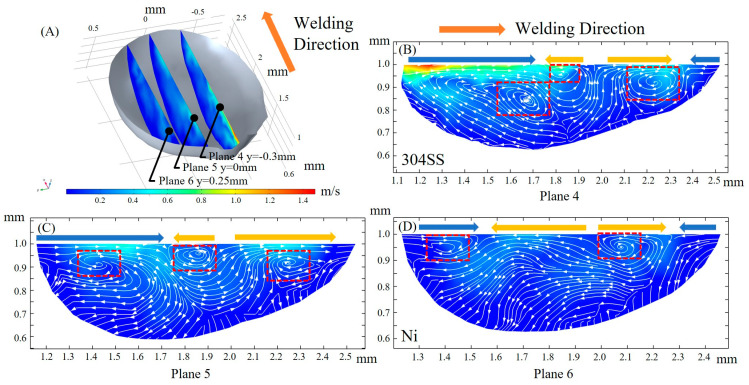
Fluid flow in the xz cross section. (**A**) 3D view; (**B**) y = −0.3 mm; (**C**) y = 0 mm; (**D**) y = 0.25 mm.

Significant variation of the fluid flow magnitude in the welding direction between the 304SS side and the Ni side is shown at three xz cross sections in Figure 10A, with the intensity of the convection gradually decreasing from plane 4 to 5 to 6 after the full development of the molten pool. The collision of inward and outward convection as a result of the complex convection caused by the transformation of the TCST due to elemental sulfur is illustrated in planes 4, 5, and 6, with two or three vortexes observed. The fluid flow at plane 4 consists of two separate vortexes, and the rear flow intensity is greater than the front, indicating that the dilution of the solute on the 304SS side occurs mainly at the rear of the molten pool. In plane 6, the fluid flows mainly from the front of the molten pool to the rear, which indicates that the solute on the Ni side is primarily conveyed from front to rear and that the outward convection is stronger than the inward convection.

**Figure 11:**
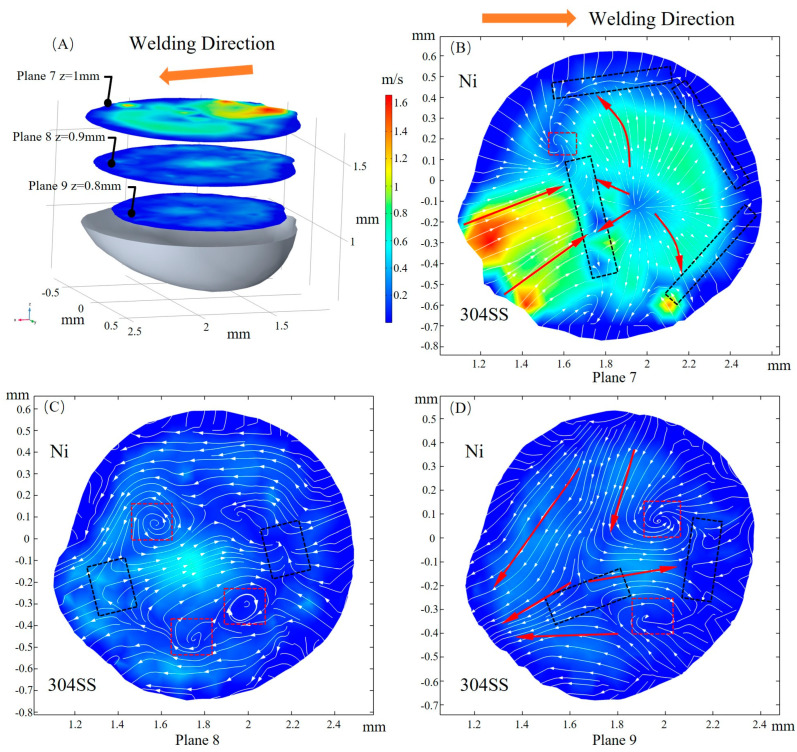
Fluid flow in the xy cross section. (**A**) 3D view; (**B**) z = 1 mm; (**C**) z = 0.9 mm; (**D**) z = 0.8 mm.

The velocity magnitude of fluid flow at three xy cross sections is plotted in Figure 11A. At plane 7, inward and outward convection collide at the rear of the laser beam (x = 2 mm, y = 0 mm) due to changes in the TCST caused by local temperature and sulfur concentration, which facilitates the mixing of solute. The flow velocity gradually decreases along the depth direction, and the convection direction changes significantly. The flow direction, identified by arrows in Figure 11B,D, at plane 7 is almost opposite to that at plane 9, and the transition of flow direction occurs at plane 8. Multiple regions of convective collisions and vortexes are marked by black and red rectangles, respectively. It is a result of complex Marangoni convection when considering the sulfur-induced transition in driving forces and flow patterns.

### 3.4. Mass Transfer

In order to study the transportation of the alloy elements, a detailed analysis of the dilution phenomenon in different planes is performed. As the molten pool develops sufficiently, mass transfer is mainly controlled by fluid flow, and the effect of diffusion is negligible. Therefore, the mass transfer characteristic is discussed with the flow characteristic in this section. The planes selected in this section are the same as those in Section 3.3.

**Figure 12:**
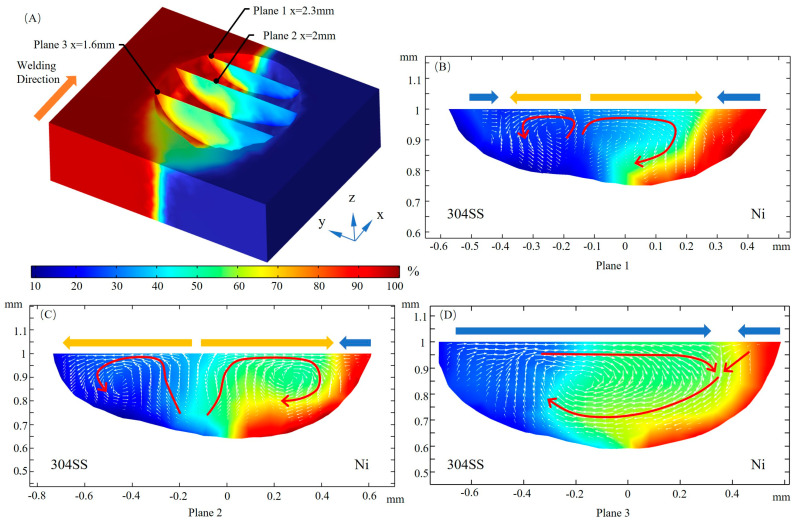
Ni concentration on yz sections. (**A**) 3D view; (**B**) x = 2.3 mm; (**C**) x = 2 mm; (**D**) x = 1.6 mm.

Figure 12 shows the distribution of Ni elements on yz cross sections, expressed as weight fractions. From Figure 12A, it can be seen that the dilution of the solute gradually becomes uniform from planes 1 to 2 and then to plane 3. For planes 1 and 2, the transport of alloying elements in the molten region around the laser beam is dominated by the branch flow from the 304SS side to the Ni side, as indicated by the red arrow in Figure 12B,C. Due to the high temperature, the negative value of the TCST makes the convection in the front of the molten pool predominantly outward, and the region on the 304SS side near the center plane y = 0 is mainly involved in the mixing and dilution of the solute on the Ni side. The rear part is in the liquid phase longer, and the elements are more evenly distributed. In this region, the convection direction changes inward, and the collision of two inward convection streams promotes the dilution of the alloying elements. Here the dilution of the Ni element to the 304SS side is more significant, as shown in Figure 12D.

**Figure 13:**
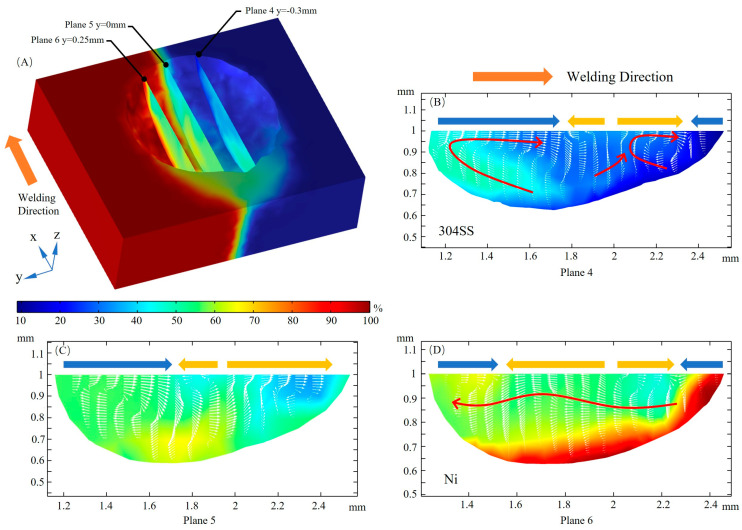
Ni concentration on xz sections. (**A**) 3D view; (**B**) y = −0.3 mm; (**C**) y = 0 mm; (**D**) y = 0.25 mm.

Figure 13 shows the mass distribution on three xz sections. For plane 4, the weight fraction of Ni in the front is less than 30%, indicating that the position slightly away from the center plane is less involved in the dilution on the Ni side. The Ni element on the 304SS side mainly comes from the rear of the molten pool and is transported from the bottom to the top. On planes 5 and 6, the weight fraction of the Ni is smaller at the front edge compared to other locations. This is an indication of the solute transfer from 304SS to Ni. For plane 6, the solute transported from the 304SS side, which is reflected by a low Ni mass fraction, is transferred from front to back, as indicated by the red arrow in Figure 13D.

**Figure 14:**
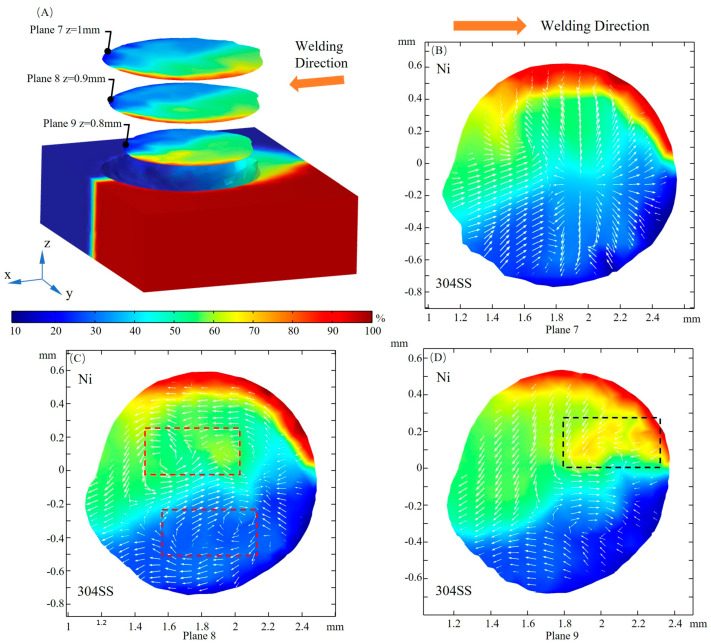
Ni concentration on xy sections. (**A**) 3D view; (**B**) z = 1 mm; (**C**) z = 0.9 mm; (**D**) z = 0.8 mm.

Figure 14A illustrates the dilution characteristics on xy sections, and for comparison, the planes are translated into their spatial position along the z-direction. For plane 7, the lateral convection across the two materials at the front of the molten pool causes the solute to mix uniformly, whereas the inward convection in approximately the same direction as the welding direction causes the solute to be in a gradient distribution state. Plane 8 is divided into two regions by the line, with a mass fraction of 40% for elemental Ni. The presence of two vortexes marked by the red dotted rectangles makes the two regions internally uniform but unable to fully transfer melted materials to each other. In plane 9, the fluid flow is predominantly front-to-back, resulting in inadequate dilution. The enrichment of elemental Ni appears at the position marked by the black square in the vicinity of a vortex. This is due to the weak flow in plane 9, where the velocity is less than 0.2 mm/s, as shown in Figure 11D.

**Figure 15:**
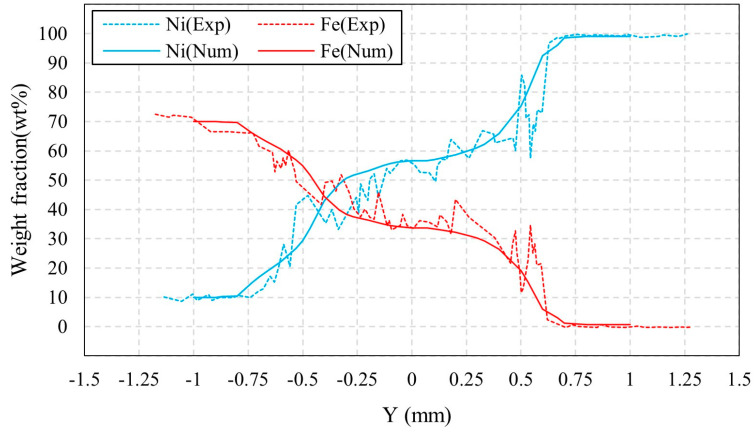
Comparison between simulated and experimentally measured [7] concentration distributions. Parameters: laser radius of 0.57 mm, welding speed of 30 mm/s, and laser power of 800 W.

In Figure 15, the element distribution of Ni and Fe at the top of the yz cross section is compared with that measured by EDS. It shows a good agreement between simulated and experimentally measured element distributions. It can be seen from Figure 15 that the intersection of curves for Ni content and Fe content is located to the left of Y = 0. On one hand, the Ni content on the Ni side is higher than the Fe content on the 304SS side, resulting in a greater amount of Ni being transported towards the 304SS side. On the other hand, for laser welding of dissimilar metals, the molten pool is asymmetric due to the difference in thermal-physical properties between the two materials. The weld pool on the 304SS side is wider than on the Ni side, as shown in Figure 12. This facilitates the intersection point of two curves on the 304SS side, that is, to the left of Y = 0.

From the above results, the mechanism of surface-active elements in base metals affecting surface tension and fluid flow is explained. It provides a theoretical basis for controlling the composition distribution, and hence, the microstructure and weld properties by varying the content of surface-active elements.

## 4. Conclusions

In this study, the relationship between the thermal behavior, driving forces, fluid flow, and mass transfer under the influence of sulfur are studied using an improved 3D numerical model. Some important conclusions are summarized as follows:

1. The molten pool is fully developed after 45 ms under the influence of sulfur, and the aspect ratio is stabilized at about 0.3. The maximum flow velocity is 1.7 m/s, and the Peclet number reaches 400 on the 304SS side and 60 on the Ni side, indicating the predominance of convective heat transfer.
2. After the molten pool reaches a quasi-steady state, sulfur is uniformly mixed in the front of the molten pool, and eventually there is a gradient distribution at the rear. The temperature-gradient term of surface shear stress plays a major role in welding direction. In the transverse direction, the temperature-gradient term of the surface shear stress is in the same order of magnitude as the concentration-gradient term, and they jointly determine the direction of inward convection. The spatial distribution of the surface-active element and temperature leads to differences in the TCST distribution. For the Ni side, the sign of the TCST shifts at 2100 K, and for the 304SS side, the sulfur element in the base metal is much higher than Ni, making the sign of the TCST shift at 2200 K. These differences further affect the fluid flow.
3. When considering the role of elemental sulfur, the velocity of inward convection is significantly higher at the rear than the other positions of the molten pool. Inward convection collisions with outward convection inside the molten pool, along with the presence of complex vortices, are conducive to solute dilution. From the front part of the molten pool to the rear, the mixing of the solute gradually becomes homogeneous, and the rear of the molten pool is an important area for sufficient dilution of solute elements, e.g., Ni, Fe, and Cr.

## Figures and Tables

**Table 1:** Chemical composition of 304SS (weight fraction (%)).

Cr	Ni	Mn	Si	C	Fe
20	10	1.32	0.83	0.08	Bal.

**Table 2:** Thermophysical properties of 304SS and Ni [17].

Parameter	304SS	Ni
Solidus temperature (K)	1672	1730
Density of solid metal (kg/m^3^)	7450	8900
Thermal conductivity of solid (W/m·K)	19.2	60.7
Specific heat of solid (J/kg·K)	711.28	515
Liquidus temperature (K)	1727	1735
Density of liquid metal (kg/m^3^)	6910	8880
Thermal conductivity of liquid (W/m·K)	50	150
Specific heat of liquid (J/kg·K)	836.8	595
Heat of fusion (kJ/kg)	272	290
Dynamic viscosity (kg/m·s)	6.70 × 10^−3^	3.68 × 10^−3^
Liquid volume thermal expansion (K^−1^)	1.96 × 10^−5^	4.50 × 10^−5^
Liquid volume concentration expansion (K^−1^)	0.078	0.078
Effective mass diffusivity (m^2^/s)	7 × 10^−7^	7 × 10^−7^

**Table 3:** Parameters used in the sub-model of surface tension [21,23].

Parameter	Fe-S	Ni-S
*γ_p_* (N/m)	1.943	1.845
*A* (N/m·K)	4.3 × $10^{-4}$	4.3 × $10^{-4}$
*Γ_S_* (mol/m^2^)	1.3 × $10^{-5}$	1.5 × $10^{-5}$
*k_l_*	0.00318	0.00318
Δ*H*^0^ (J/mol)	−1.88 × $10^{5}$	−1.47 × $10^{5}$

**Table 4:** Welding parameters used in the simulation.

Parameter	Value
Welding speed (mm/s)	20, 30
Laser power (W)	800
Laser spot (mm)	0.57
Ambient temperature (K)	300
Laser absorption efficiency	0.26
Stefan-Boltzmann constant (W/$m^{2}K^{4}$)	5.67 × $10^{-8}$
Emissivity	0.2
Convective heat transfer coefficient at top surface (W/$m^{2}K$)	100

## Data Availability

Not applicable.
